# Survey data on Vietnamese propensity to attend periodic general health examinations

**DOI:** 10.1038/sdata.2017.142

**Published:** 2017-10-03

**Authors:** Quan-Hoang Vuong

**Affiliations:** 1 Western University Hanoi, Centre for Interdisciplinary Social Research, Yen Nghia Ward, Ha Dong District, Hanoi, 100000, Vietnam

**Keywords:** Developing world, Population screening

## Abstract

As general living standards rise, so does the demand for periodic general health examinations (GHEs). Research on the subject, however, has reached opposing conclusions on the value of GHEs, although methodological limitations in previous works make these differences hard to resolve. Here, we present data from a socio-demographic survey of behaviours and tendencies concerning periodic GHE attendance in Vietnam. These data are shown to be suitable for evaluating the impact of demographic and socio-economic elements on regular health examinations. By presenting the methods used in this survey and by describing the enquiries mentioned in the dataset, this article aims to promote data-collecting methodologies that can help policy-makers and health communicators derive practical conclusions.

## Background & Summary

Periodic general health examination (GHE) programmes emerged a long time ago but it was not until the 20th century brought increases in income and living standards that they began to attract attention and interest^1^. They attracted interest because there are substantial, sometimes inappropriate charges for both preventive and medical treatment services^2–5^ and these charges pose a substantial obstacle to people who wish to have regular health check-ups^6^. In addition, a number of people remain skeptical about the value of periodic GHE, either finding them costly and without benefit^7–9^ or questioning their quality^10,11^. Vietnamese patients are sometimes even skeptical of health professionals’ expertise^12^. Unsurprisingly, therefore, many have suggested replacing periodic health examinations with more effective healthcare solutions^13,14^.

That said, the benefits of GHEs should not be overlooked. Regular medical checks provide individuals with updates on their health status and symptoms^15^, making it easier to detect illnesses at an early stage and seek suitable treatment^16^. Studies have shown that periodic GHEs help to detect and combat breast and ovarian cancers^16,17^. In addition, GHEs may also lower the cost of future treatment^18^, and help to reduce economic inequality^19^. Thus, periodic GHEs can be considered genuinely necessary^6^.

The decision about whether or not to attend regular GHEs depends on multiple factors. The influence of one factor, possession of health insurance, remains subject to debate. Meer and Rosen^20^ reported that insured patients tended to have GHEs more often^20^, however Lurie *et al.* argued that health insurance only encourages people to take advantage of medical services and fails to improve public health^21^.

A review of the literature revealed that many authors have analyzed the effect of patient attitudes to the time, cost and quality of the medical service provided on frequency of GHEs, with conflicting results. These studies are, however, affected by certain limitations. For example, some studies had restricted samples or relied on limited, biased data provided by companies and goverments^22,23^ and others were based on postal surveys^24^. All these issues may limit the technical validity of the data. In addition most previous research on GHEs was limited to simple descriptive statistics and group comparisons. Although these analyses have produced valid results and insights they are not suitable for evaluating interactions between variables, which is necessary if we wish to learn more about the relationships between groups of socio-demographic, psychological, economic and socio-cultural factors and their influence on attitudes and behaviour in relation to GHEs^23,25^. The limitations of the earlier research can be addressed by using an appropriate research design and analytical techniques, such as multiple logistic regression including both continuous and discrete variables. This approach enables a wider evaluation and the testing of more hypotheses but does not require strict assumptions about probability mass/density distributions. One of the chief benefits of logistic regression is that estimates of odds ratios, an important measure of association, can be obtained from parameter estimates^26^. Insights obtained from a GHE survey should not be limited to general trends or confirmation of associations between variables because by using estimated coefficients, the computing of conditional probabilities for certain events under specific conditions^27^ will provide information with important policy implications.

We therefore conducted a survey to explore behaviour and attitudes to GHEs in a developing country, namely Vietnam. Our aims were to determine whether our survey data corroborated previous findings on GHEs in Vietnam and to provide additional information of practical significance. The dataset is particularly important due to the recent rise in concern about cancers and other diseases, such as diabetes and HIV.

## Methods

Our data were gathered through an interview-based survey of behaviour and attitudes to GHEs amongst inhabitants of Hanoi and Hung Yen, Vietnam. Interviews were conducted face-to-face and data were recorded on paper. The idea of this survey was based mainly on several previous studies of the effects of medical costs on patients’ lives after treatment^28,29,30^. One study showed that patients, especially poor patients who had borrowed money to pay for treatment, tended to fall into destitution after receiving hospital treatments^28^. Many desperate patients had little choice but to live together and support each other as they struggled to earn a living and pay for prolonged treatment^29,30^. This evidence about the harsh reality of the situation facing seriously ill patients led to recognition that prevention and early detection of disease are critically important.

The project consists of five phases: (1) Questionnaire design; (2) Face-to-face interviews; (3) Quality control for questionnaire answers; (4) Preparing the dataset; (5) Data analysis.

### Survey sample

Participants were chosen at random. All mentally competent residents in survey locations were invited to take part. Interviews did not begin until potential participants had been given information about the institutions responsible for the research, the objectives of the research and the methods of analysing the data, and had agreed to take part. Participants have been informed of indirect identifiers in the dataset and have consented to public use of their personal information under the condition that their names must be removed. The dataset—with respondent names being removed—is thus suitable for open access.

### Survey design

The survey was conducted between September and November 2016 in locations such as secondary schools, hospitals, companies, government agencies and randomly selected households in Hanoi, including Hospital 125 Thai Thinh (Dong Da District) and Vietnam-Germany Hospital (Hoan Kiem District). The survey team consisted of seven key members who were associated with Vuong & Associates research office and a dozen assistants. Key members wore identification badges in the field.

Interviewers recorded the time taken for each interview. The numbers of refusals and acceptances were reported at the end of each day and summed at the conclusion of the fieldwork.

The survey team adhered to the ethical code of the institutions responsible for the research. All questionnaires were checked and their validity confirmed by the team member who collected them and the team supervisor. Access to the database is open to the public, following the agreement between participants and the research team.

### Survey validation

Before the interview respondents were given instructions on the response formats for the various questions, for example to choose only one answer when the question required selection of the most appropriate response amongst multiple choices. For questions where responses were to be given using a numerical scale the interviewer ensured that the respondent understood the scale and gave a score within the allotted range. In addition, all collected questionnaires were checked three times to ensure the quality and validity of the data: when the interviewer returned to the team, when data were entered into the database and before exploratory analysis.

### Data collection

**Figure 1:**
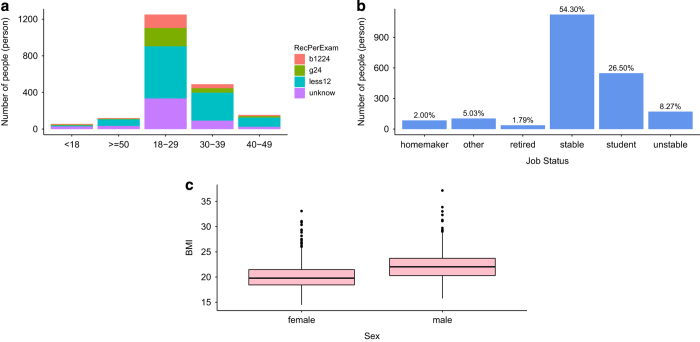
Histograms of participants. By age (**a**), and job status (**b**), and distribution of BMI values by sex (**c**) Fig. 1a shows the distribution of people by age, and within each age group the distribution of times since last GHE. (**b**) shows the distribution of participants by job status. (**c**) presents the distribution of average BMI by sex and indicates 25 and 75% quartiles.

A total of 2,479 people were approached, of whom 409 refused to take part. The total number of observations was thus 2,070, two of which were invalid and excluded from analysis, yielding a final sample of 2,068 valid responses. On average, one out of six people refused to take part when invited to do so. Interviews lasted for approximately 12–15 min. Participants were male and female and ranged in age from 13 to 83 years. The female participation rate was 64.08% (1,340/2,068). The average age of participants was 29.17 years (*s.d.=*10.09, 95% CI: 28.74–29.60). The majority of respondents (60%, 581/2,068; see Fig. 1a) were aged between 18 and 30 years old. Most respondents had had their last GHE less than one year before the date of the interview. The majority of the sample was married (57.35%) and 54.35% of respondents had a stable job (Fig. 1b).

Participants provided us with their weight and height to enable us to calculate their body mass index (BMI). Most participants had a relatively healthy BMI (*M=*20.848, *s.d.*=2.67, 95% CI: 20.73–20.96). On average, male respondents had a higher BMI than female respondents (Fig. 1c).

### Data and materials

Data were used to analyse patterns in GHE engagement and to assess how specific variables influenced GHE behaviour. Time since last GHE was used as the dependent variable in analyses of factors affecting the frequency with which individuals attended medical checks.

#### Materials

The raw data were first entered into a MS Excel file, then converted into ‘comma-separated values’ (CSV) format (which can be found at 11102016Med4.csv [Data Citation 1]). Data were analysed in R (3.3.1). Estimates were calculated using the baseline-categorical logit model (BCL)^27^.

As most variables were categorical and most data for response and predictor variables were discrete we used a logistic model. Logistic models are used to predict the probability of each value of the dependent variable given specific values of the independent variables.

The general equation for the baseline-categorical logit model is:

$$\ln[\pi_{j}\left(x\right)/\pi_{J}\left(x\right)]=\alpha_{j}+{\beta_{j}}^{T}x,\;j=1,\ldots ,\;J-1.$$

where **x** is the independent variable; and π_*j*_(**x**)=P(*Y*=*j*|**x**) its probability. Thus π_*j*_=P(*Y*_*ij*_=1), with *Y* as the dependent variable.

In the logit model under consideration, the probability of an event is computed as:

$$\pi_{j}\left(x\right)=\exp(\alpha_{j}+{\beta_{j}}^{T}x)/[1{+}^{J-1}\sum_{h-1}\exp(\alpha_{j}+{\beta_{j}}^{T}x)]$$

Beta coefficients can be regressed directly from the original CSV file. In this case, the reference independent variable’s categories will be set by default. Reference categories cannot, however, be modified by the analyst. Therefore, we perform regression on distribution tables of the sample, in CSV format. File tab4.1.csv [Data Citation 1] is an example of such a table.

We also used linear regression or ordinary least square (OLS) analysis for the numerical variables. The general equation for the OLS analysis is as follows:

$$Y=\alpha +\beta_{1}X_{1}+\beta_{2}X_{2}+\ldots +\beta_{k}X_{k}$$

*Y* is a continuous variable; the independent variables *X*_*i*_ can be concrete, categorical or continuous.

#### Response coding

Both questions and participants’ responses were codified into variables and variable categories in our dataset. The demographic variables were as follows: ‘sex’ (male; female), ‘age’, ‘weight’ (in cm) and ‘height’ (in kg). Because the participants were recruited randomly and fieldwork was carried out in a variety of locations it was not practical to measure participants’ height and weight directly, so respondents were asked to provide their most recent measurements of height and weight. Most Vietnamese people memorize their height and weight, as a considerable number of administrative procedures in the country require personal documents for which these measurements are indispensable. In addition it is not complicated to take measurements of one’s height and weight as electronic devices and mobile phone apps for doing so are widely available and fairly easy to use. For these reasons we consider the data provided by respondents to be reliable. From them we calculated BMI, using the formula BMI=weight/(height×height).

Marital status is referred to as ‘MaritalStt’ (married; unmarried; other). Job status was captured as the variable ‘JobStt’ (stable; unstable; student; retired; homemaker; other). Educational attainment was captured as ‘Edu’ (‘PostGrad’ (post-graduate); ‘Grad’ (college/university); ‘Second’ (high school); ‘Hi’ (middle school)). Health insurance status was represented by a binary variable, ‘HealthIns’. Questions concerning weight, height and BMI also appear in the questionnaire.

The variables were time since last medical examination (‘RecExam’) and time since last GHE (‘RecPerExam’) and both were coded as follows: ‘less12’=less than 12 months; ‘b1224’=between 12 and 24 months; ‘g24’=over 24 months; ‘unknown’=respondent unable to recall. Before respondents answered the relevant questions the interviewer carefully explained the difference between them (and made sure the respondent understood the questions properly, in order to ensure that responses were accurate. ‘Time since last medical examination’ is the length of time since the respondent last visited a doctor with symptoms of disease, whereas ‘time since last GHE’ is the length of time since the respondent’s last GHE. GHEs are conducted periodically regardless of whether an individual has any signs of illness or disease and are intended to track individuals’ health status and detect disease at a pre-symptomatic stage. During a GHE, people will receive a list of tests, including clinical examinations and subclinical tests, such as diagnostic imaging and functional exploration.

*Reasons for their most recent GHE*, captured in the variable ‘RecExam’ were coded as follows: ‘noti.disease’=concerns over illnesses/epidemics; ‘adv.sig’=worrying symptoms; ‘request’=prompted by employer/community/insurance; ‘volunteer’=no immediate reason. We also collected data on how often respondents believed GHEs should be carried out: every 6 months (‘6 m’); every 12 months (‘12 m’), every 18 months (‘18 m’) or less than every 18 months (‘g18m’).

One question dealt with reasons why people might hesitate to take a GHE. Binary yes/no responses to the following reasons were solicited: GHE is a waste of time (‘Wsttime’); GHE is a waste of money (‘Wstmon’); fear of discovering diseases (‘DiscDisease’); little faith in the quality of the medical service (‘Lessbelqual’); do not consider GHEs to be urgent or important (‘NotImp’). A similar format was used to explore reasons for attending a GHE, with options as follows: health is first priority (‘HthyPriority’); GHEs are subsidized by employer/community (‘ComSubsidy’); have acquired the habit of regular GHEs from family/employer (‘Habit’); constantly follow updates on their health measures (‘FlwHealth’).

To gain more insight into the health status of respondents and their families we asked participants whether they or a member of their family were receiving long-term medical treatment (‘PerTrmt’ and ‘AcqTrmt’ respectively; binary responses). We also asked respondents whether they and their family all enjoyed good health ‘StabHthStt’; binary response: ‘yes’ if respondent *and* family all in good health, otherwise ‘no’). This question was used to evaluate the extent to which family members’ health status is related. Finally we asked what participants’ preferred way of dealing with new symptoms (StChoise) would be, the options were: ‘clinic’=go to the clinic and consult professionals; ‘askrel’=seek advice from family and relatives; ‘selfstudy’=do personal research.

We assumed that individuals’ attitude to health would be correlated with possession of common items of medical equipment and the ability to use them, so we asked the following questions: (1) Do you keep a medical cabinet and basic medical equipment in your house? (‘MedCabinet’); (2) Do you have the skills to use basic medical equipment? (‘Tooluseskill’); (3) Do you have experience in taking care of a sick family member? (‘ExpCare’); (4) Does your family regularly take simple medical measurements (blood pressure, eye sight, weight etc.)? (‘ExamTools’).

We assessed perceptions of the quality of periodic GHE sessions using five questions to which responses were given using a continuous, 1 to 5 scale (1=lowest quality). The variables were as follows: ‘Tangibles’=quality of medical equipment and personnel; ‘Reliability’=ability of examiner to perform medical services that meet the patient’s expectations; ‘Respon’=timeliness of service; ‘Assurance’=knowledge/ability to assure professional reliance; ‘Empathy’=thoughtfulness and having a high sense of responsibility. We also asked participants to tell us there general opinion of public health (‘CHPerc’), the options were: ‘good’, ‘quite good’, ‘bad’ and ‘unknown’.

Cost of treatment is one of the most important factors in people’s decision of having GHEs. Cost can influence whether patients go to the hospital or clinic for health checks, particularly if they do not experience signs of illness. In the survey, GHE costs are divided into three categories: ‘low’=under 1 million VND; ‘med’=from 1 to 2 million VND; ‘hi’=over 2 million VND. Respondents were also asked which of the following options they would choose if they were provided cash for having GHEs (‘Usemon’): use all the money to have a GHE soon (‘allsoon’); use part of the money for a GHE and save the rest (‘partly’); take the money and have a GHE later (‘later’).

Information in the mass media on health care in general, and on GHEs in particular, can also affect attendance at periodic medical examinations and judgments of medical service quality. We therefore asked participants to evaluate several aspects of the information they had received on GHEs, using a 1 to 5 scale: sufficiency (‘SuffInfo’); attractiveness (‘AttractInfo’); impressiveness (‘ImpressInfo’); popularity (‘PopularInfo’).

Development in science and technology mean that the use of information technology (IT) in subclinical diagnosis is becoming more and more widespread. At present there is only limited use of IT to support healthcare in Vietnam, for example healthcare queuing apps and more complex applications such as online consultation, diagnostic imaging, remote health treatment, electronic medical records etc. Not everyone is ready to accept the use of IT to support diagnostic assessment. We assessed such readiness using two questions: (1) ‘Are you willing to use IT to detect health problems if it is reliable’ (‘UseIT’) and (2) ‘If a healthcare app indicated that you needed to have a GHE would you actually arrange one?’ (‘AfterIT’).

At the end of the questionnaire there were two questions about participation in sports and physical exercise that were used to evaluate attitude to sports and perception of the health benefits of regular exercise: (1) ‘How much time do people need to spend on sports and physical exercise to stay in shape?’ (‘SuitExer’) and (2) ‘How much time do you spend on sports and physical exercise?’ (‘EvalExer’). Response options for the second question were ‘more than enough’ (‘verysuff’); ‘enough’ (‘quitesuff’); only a little (‘little’); ‘none or almost none’ (‘trivial’).

Measurement of the dependent variable and the control variable. The code used in R(3.3.2) was:

   
> model4.1=read.csv(‘D:/.../tab4.1.csv’,header=T)
> attach(model4.1)
> fit.model4.1=vglm(cbind (unknown,g12,less12)~Wsttime +Wstmon+HthyPriority+FlwHealth +HealthIns,data=model4.1,family=multinomial)
> summary(fit.model4.1)


These commands were intended to determine how the length of time since an individual’s most recent GHE is related to possession of health insurance, concerns that GHEs are a waste of time and money, prioritisation of health and regular following of health updates. The results are presented in Table 1.

The model’s fitness test was conducted to verify that all the coefficients are not equal to zero simultaneously, that is the null hypothesis H_0_: β_1_=β_2_=...=0, yields the *P*-value:

p=1−pchisq(2×(−151.22+249.91),10)≈0

with df=(62–52)=10 (see Agresti)^31^. Thus, H_0_ was decisively rejected.

**Figure 2:**
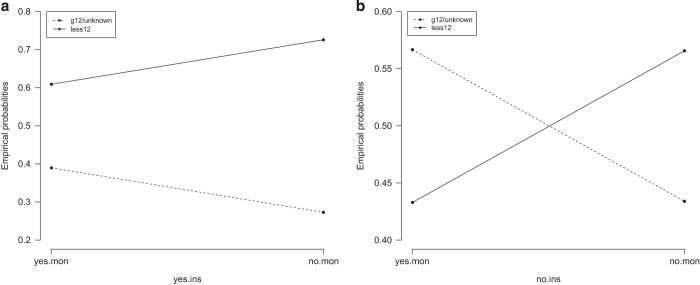
Shows the difference between insured and uninsured patients with respect to likelihood of attending a GHE in the near future. The two graphs are constructed using conditional probabilities calculated from the estimated coefficients presented in Table 1. The method of calculation was as described by Agresti^27^. From (**a**,**b**) the shifting trends of empirical probabilities are similar for both insured and uninsured patients. However, the changes in numerical probabilities are significantly different, and in (**b**), the two probability lines intersect, at about 50%. So 50% can be seen as a probability threshold where uninsured patients are indifferent in their decisions to have a GHE in the near future or not. There exists no such threshold for insured patients as seen in (**a**).

The data in Table 1 were used to calculate conditional probabilities, which provide some useful remarks: (i) if there are no financial or temporal constraints people will attend GHEs to try to ensure early detection of diseases and timely treatment; and (ii) possession of health insurance is positively associated with attendance at GHEs, even in the case of people in financial difficulties (Fig. 2).

On the basis of these results we suggest that attendance at GHEs could be improved by increasing the budget for supported healthcare schemes, raising the actual coverage of health insurance and improving the quality of medical services offered to people with health insurance.

### Code availability

Data were analysed using the statistical software R (release 3.3.1). The code used in the analyses is available as a pdf file (Supplementary File 1) which includes examples of code used to read the input data, create contingency tables and carry out multiple logistic regression for the dependent variable ‘RecPerExam’ and predictor variables ‘Wsttime’, ‘Wstmon’, ‘HthyPriority’, ‘FlwHealth’ and ‘HealthIns’.

**Figure 3:**
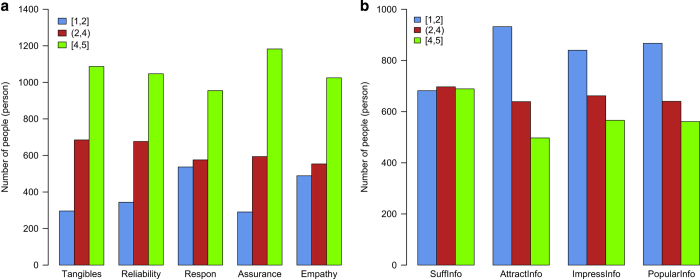
Distribution of participants’ perceptions of GHE. Based upon perception of GHE service quality (**a**) and perception of mass media information on GHEs (**b**). Symbols in the figure are presented in Answer Coding.

The R code for generating Figs 1–3 is also included.

## Data Records

Files are in.csv format, both for conversions of the original Excel data and computed frequencies used in regression models (Data Citation 1).

## Technical Validation

Data were computerized by two specialists from our research team: one person entered the data into an MS Excel file and the other checked the file to ensure that the recorded data accurately represented the responses recorded on paper questionnaires. In cases where there was doubt about the nature of a participant’s response we contacted the surveyor to check the response.

The logistic regression model in the example was assessed in terms of the statistical significance of its coefficients. As shown in Table 1, the majority of coefficients have *P*<0.05, except the intercepts and the coefficients of ‘HthyPriority’ in the equation logit(unknown|less12). The null hypothesis was rejected, therore it can be inferred that there are correlations between the aforementioned independent and dependent variables.

Also, odds ratios can be useful in analyzing the survey data. The largest odds ratio was that for ‘Wsttime’=‘yes’ in the logit equation of (unknown|less12) (1.939), indicating that, amongst the investigated variables, ‘Wsttime’ had the most powerful influence (positive) on the probability of ‘RecPerExam’=‘unknown’.‘HealthIns’=‘yes’ had the smallest odds ratio (0.477), representing the declining probability of ‘RecPerExam’=‘unknown’. ‘HthyPriority’=‘yes’ had an odds ratio ~0.9, nearly 1, indicating that prioritising health had little effect on the dependent variable.

### Descriptive statistics

Table 2 describes some of the categorical variables in the dataset. Over half the sample (*n=*1,059, 51.21%) had had a GHE less than a year ago. One of the most common reasons given for hesitating to have a GHE was that they are waste of time; nearly 52% of participants who were reluctant to attend GHEs mentioned this as a reason. Amongst those who were prepared to attend GHEs, the main reason given was that health was a priority (81%).

If they experienced symptoms of ill-health the majority of participants would choose to go to a clinic (43.04%). Most respondents (86.32%) believed that a GHE should cost less than 2 million VND, indicating that reasonable pricing is a big concern for people in relation to periodic GHEs.

With respect to use of IT to support healthcare, 42.12% of participants claimed to be willing to use IT if it had been shown to be reliable. If a healthcare app indicated symptoms of disease then 39.41% of participants would be willing to have a GHE.

Data in the form of five-point Likert scale responses were classified into three groups: 1–1.99 points; 2–3.99 points; 4–5 points. Most respondents gave GHEs 4 or 5 points for all aspects of the quality of medical service provided (Fig. 3a). The quality factor with the lowest mean scores was timeliness (‘Respon’) (*M*=3.38, 95% CI: 3.33–3.43). On the other hand, with regards to mass media information on periodic GHEs, only informational sufficiency (‘SuffInfo’) had relatively the same number of participants in all three score groups (Fig. 3b). The remaining factors (attractiveness, impressiveness and popularity) attracted low scores (1 or 2 points) from most participants.

## Usage Notes

The dataset provides the empirical data needed to answer research questions about periodic health care behaviours, such as the identity of psychological factors affecting the timing of health check-ups, the propensity to spend on GHEs and perception of the optimal frequency of GHEs. The dataset can also be used to evaluate perceptions of GHE service quality and factors affecting such perceptions. For practical usage, media coverage and expansion of medical information regarding GHEs may also be a subject of discussion upon exploiting these data.

## Additional information

**How to cite this article:** Vuong, Q.-H. Survey data on Vietnamese propensity to attend periodic general health examinations. *Sci. Data* 4:170142 doi: 10.1038/sdata.2017.142 (2017).

**Publisher’s note:** Springer Nature remains neutral with regard to jurisdictional claims in published maps and institutional affiliations.

## Supplementary Material



Supplementary File 1

## Figures and Tables

**Table 1:** Estimated coefficients

	**Intercept**	**‘Wsttime’**	**‘Wstmon’**	**‘HthyPriority’**	**‘FlwHealth’**	**‘HealthIns’**
		**‘yes’**	**‘yes’**	**‘yes’**	**‘yes’**	**‘yes’**
	**β**_**0**_	**β**_**1**_	**β**_**2**_	**β**_**3**_	**β**_**4**_	**β**_**5**_
logit(unknown|less12)	−0.053 [−0.306]	0.289* [2.343]	0.581*** [4.599]	−0.107 [−0.711]	−0.732*** [−6.242]	−0.740*** [−5.174]
logit(g12|less12)	−0.098 [−0.562]	0.662*** [5.243]	0.474*** [3.713]	−0.578*** [−3.990]	−0.354** [−2.983]	−0.686*** [−4.676]
Table 1. presents estimates obtained from the logistic regression model using ‘RecPerExam’ as the dependent variable and ‘Wsttime’, ‘Wstmon’, ‘HthyPriority’, ‘FlwHealth’ and ‘HealthIns’ as independent variables.						
Signif. codes: 0 ‘***’ 0.001 ‘**’ 0.01 ‘*’ 0.05 ‘.’ 0.1 ‘ ’ 1; z-value in square brackets; baseline category for: ‘Wsttime’=‘no’; and, ‘Wstmon’=‘no’, ‘HthyPriority’=‘no’, ‘FlwHealth’=‘no’, ‘HealthIns’=‘no’. Log-likelihood: −151.2242 on 52 degrees of freedom. Residual deviance: 91.2185 on 52 degrees of freedom.						

**Table 2:** A few basic statistical indicators

**Characteristics**	***N***	**Percentage (%)**
*Health insurance*		
Yes	1,700	82.21
No	368	17.79
*Time since most recent GHE*		
Under 12 months	1,059	51.21
12 months or more	493	23.84
Unknown (not recalled)	516	24.95
*Hesitation due to seeing GHE as a waste of money*		
Yes	770	37.23
No	1,298	62.77
*Readiness due to health being considered top priority*		
Yes	1,675	81.00
No	393	19.00
*Readiness due to sensitivity to health matters*		
Yes	977	47.24
No	1,091	52.76
*Perception toward public health status*		
Good	337	16.30
Quite good	722	34.91
Not good, problematic	749	36.22
Unknown	260	12.57
*Affordable GHE costs*		
Less than VND 1 million	876	42.36
VND 1–2 million	909	43.96
Above VND 2 million	283	13.68
*Ready to use IT applications*		
Yes	871	42.12
Maybe	721	34.86
No	476	23.02
*Take GHE if IT applications show health problems*		
Yes	815	39.41
Maybe	900	43.52
No	353	17.07
*Evaluation of GHE service quality*		
From 1 to<2 points	60	2.90
From 2 to<4 points	1,291	62.43
From 4 to 5 points	717	34.67
Table 2. gives descriptive statistics, namely number and frequency, for categorical variables.		
