# Supplementary material for: Survey data on Vietnamese propensity to attend periodic general health examinations
Source: Sci Data. 2017 Oct 3;4:170142. doi: 10.1038/sdata.2017.142 (PMC5625553; doi:10.1038/sdata.2017.142)
Supplement: Supplementary File 1 [file sdata2017142-s2.pdf]

#Input data

```
> med4=read.csv("D:/V&A/Med4/11102016Med4.csv",header=T)
> attach(med4)
> names(med4)
#
```

#Creating a frequency table

```
>tab4.1=xtab(~Wsttime+Wstmon+HthyPriority+FlwHealth+HealthIns+ RecPerExam)
>ftable(tab4.1)
#
```

# Regression with response variable “RecPerExam” and predictors “Wsttime”, “Wstmon”, “HthyPriority”, “FlwHealth”, and “HealthIns”

```
> model4.1=read.csv("D:/V&A/Med4/tab4.1.csv",header=T)
> attach(model4.1)
>
fit.model4.1=vglm(cbind(unknown,g12,less12)~Wsttime+Wstmon+HthyPriority+FlwHealth+HealthIns,data=model4.1,family=multinomial)
> summary(fit.model4.1)
#
```

# Odds ratios

```
> exp(coef(fit.model4.1))

##
```

# Creating Figure 1

```
# Creating Figure 1a

> attach(med4)

> library(ggplot2)

> p1=ggplot(med4,aes(x=Age_gr, fill=RecPerExam))+geom_bar()+ylab("Number of people (person)")+xlab("")+theme_bw()+theme_classic()+labs(title="1A")

# Creating Figure 1b

> df <-
data.frame(job=c("stable","unstable","student","retired","homemaker","other"),freq=c(1123,171,548,37,85,104),pro=c("54.30%","8.27%","26.50%","1.79%","2.00%","5.03%"))

> p2=ggplot(data=df, aes(x=job, y=freq))+ylab("Number of people (person)")+xlab("Job Status") +geom_bar(stat="identity", fill="cornflowerblue")+geom_text(aes(label=pro), vjust=-0.3, size=3.0)+theme_minimal()+theme_classic()+labs(title="1B")

# Creating Figure 1c

> attach(med4)

> p3=ggplot(med4,aes(x=Sex,y=BMI))+ylab("BMI")+xlab("Sex")+geom_boxplot(fill = "pink", colour = "black")+theme_minimal()+theme_classic()+labs(title="1C")

# Combining multiple plots into one overall graph
```

```
> library(grid)

> grid.newpage()

> pushViewport(viewport(layout = grid.layout(2, 4)))

> print(p1, vp = viewport(layout.pos.row = 1, layout.pos.col = 1:2))

> print(p2, vp = viewport(layout.pos.row = 1, layout.pos.col = 3:4))

> print(p3, vp = viewport(layout.pos.row = 2, layout.pos.col = 2:3))

##
```

# Creating Figure 2

```
> par(mfrow=c(1,2))

#Creating Figure 2 a

> m11=c(0.390 , 0.273)

> m12 = c(0.610 , 0.727)

> plot(m11,type="o",col="black",pch=20,lty=2,ylim=c(0.2,0.8),axes=FALSE,ann=FALSE)

> axis(1,at=1:2,lab=c("yes.mon","no.mon"))

> axis(2, las=1, at=c(0.2,0.3,0.4,0.5,0.6,0.7,0.8))

> lines(m12,type="o",pch=20,col="black")

> title(xlab="yes.ins")

> title(ylab="Empirical probabilities")

>legend(1,0.8,c("g12/unknown","less12"),cex=0.8,col=c("black","black"),pch=20:20,lt
y=2:1)

> title(main="2A")

# Creating Figure 2b

> m21=c(0.567,0.434)

> m22=c(0.433,0.566)

> plot(m21,type="o",col="black",pch=20,lty=2,ylim=c(0.4,0.6),axes=FALSE,ann=FALSE)

> axis(1,at=1:2,lab=c("yes.mon","no.mon"))

> axis(2, las=1, at=c(0.40,0.45,0.50,0.55,0.60))

> lines(m22,type="o",pch=20,col="black")

> title(xlab="no.ins")

> title(ylab="Empirical probabilities")

>
legend(1,0.6,c("g12/unknown","less12"),cex=0.8,col=c("black","black"),pch=20:20,lty
=2:1)

> title(main="2B")
```

##

# Creating Figure 3

```
> par(mfrow=c(1,2))
```

```
# Creating Figure 3a
```

```
>
data=structure(list(B=c(296,685,1087),C=c(344,677,1047),D=c(537,576,955),E=c(291,59
4,1183),F=c(489,554,1025)),.Names=c("Tangibles","Reliability","Respon","Assurance",
"Empathy"),class="data.frame",row.names=c(NA,-3L))
```

```
> colours=c("cornflowerblue","firebrick","chartreuse")
```

```
> barplot(as.matrix(data), ylim=c(0, 1400),ylab = "Number of people (person)",main=
"3A", beside=TRUE,col=colours)
```

```
> legend("topleft", c("[1,2]","(2,4)","[4,5]"), cex=1, bty="n", fill=colours)
```

```
# Creating Figure 3b
```

```
>
data=structure(list(B=c(682,697,689),C=c(932,639,497),D=c(840,662,566),E=c(867,640,
561)),.Names=c("SuffInfo","AttractInfo","ImpressInfo","PopularInfo"),class="data.fr
ame",row.names=c(NA,-3L))
```

```
> colours=c("cornflowerblue","firebrick","chartreuse")
```

```
> barplot(as.matrix(data), ylim=c(0, 1000),ylab = "Number of people (person)",
main= "3B", beside=TRUE,col=colours)
```

```
> legend("topleft", c("[1,2]","(2,4)","[4,5]"), cex=1, bty="n", fill=colours)
```
